# The Processing of Object Identity Information by Women and Men

**DOI:** 10.1371/journal.pone.0118984

**Published:** 2015-03-10

**Authors:** Michael Tlauka

**Affiliations:** Flinders University, Adelaide, Australia; University of Melbourne, AUSTRALIA

## Abstract

The study examined whether women excel at tasks which require processing the identity of objects information as has been suggested in the context of the well-known object location memory task. In a computer-simulated task, university students were shown simulated indoor and outdoor house scenes. After studying a scene the students were presented with two images. One was the original image and the other a modified version in which one object was either rotated by ninety degrees or substituted with a similar looking object. The participants were asked to indicate the original image. The main finding was that no sex effect was obtained in this task. The female and male students did not differ on a verbal ability test, and their 2D:4D ratios were found to be comparable.

## Introduction

Sex differences in spatial ability have been widely reported in the literature on human spatial cognition [1, 2]. Males have been observed to outperform females on a variety of spatial tasks such as navigation and mental rotation [3–6]. Sex differences in brain activation patterns have also been found, with Jordan, Wüstenberg, Heinze, Peters, and Jäncke [7] obtaining differential cortical activation patterns in males and females engaged in mental rotation tasks [8].

Intriguingly, there is only one documented spatial task, object location memory, in which females have been shown to display superior ability by comparison with their male counterparts [9–13]. In the object location memory test, participants are commonly presented with a two-dimensional array of objects. After learning the array the participants are shown a second array in which some objects have swapped positions (object exchanges). It is the participants’ task to identify which objects have changed positions. Females have been found to identify more object exchanges than males [11]. According to Silverman and Eals [12] selection for successful food gathering in small home ranges is the origin of better performance on the object location memory task by females [14, 15].

James and Kimura [16] introduced an interesting variation of the object location memory task. In addition to the object exchange task they include an *object shift* condition in which approximately half of the objects were moved to previously unoccupied locations. A sex effect favouring women was observed for object exchanges, but not for object shifts (but see [17]). According to James and Kimura [16] their results suggest that women excel on spatial tasks that depend on the identification of objects (as measured by the object exchanges) rather than on memory for object locations (as measured by object shifts).

The differentiation between object identity and object location information has found support in the relevant literature [18, 13]. Voyer et al. [13] distinguish between three components in object location memory: object processing, spatial-location processing and the binding of objects to locations. The present investigation focuses on the first component (identity information) and examines James and Kimura’s [16] claim that women’s ability to process object identity is superior to that of men.

In the present study, University students studied simulated indoor and outdoor house scenes (e.g., kitchens, bedrooms, gardens) displayed on a computer screen. After studying the initial scene the students were presented with two images, either the original scene or another scene which differed from the original scene. The participants were asked to indicate the original image. Note that this procedure tested the students’ knowledge of object identity since the objects were always presented in the same location.

This investigation differed from James and Kimura [16] principally in two ways. First, while the stimuli used by James and Kimura were artificial (comprising semantically unrelated objects such as a teapot, elephant etc.), the use of indoor and outdoor scenes here allowed an examination of object identity effects in the context of more realistic stimuli which people encounter in their daily lives. Given that sex differences have often been explained with reference to evolutionary accounts [19, 20], these effects should be observable across a wide range of stimuli. In other words, if object identity is a relevant factor contributing to sex differences, these effects should not be confined to the mostly artificial stimuli employed in earlier work [21].

The study differed from previous work also in that it included 2D:4D ratios. The term digit ratio is used for the proportion in length between the second and fourth digits of the human hand (the index and ring fingers, respectively). In males, the second digit tends to be shorter than the fourth, resulting in a lower ratio. In females, the two digits tend to be of similar length, resulting in a higher ratio [22].

Spatial ability is influenced by prenatal concentrations of sex hormones which are critical for the development of spatial skills. According to Geschwind and Galaburda [23] prenatal testosterone levels influence the development of the right hemisphere thereby leading to improved visuo-spatial abilities. Critically, 2D:4D ratios have been found to be negatively associated with prenatal testosterone [24–26] such that lower 2D:4D ratios are associated with increased prenatal testosterone levels [27].

To summarise, James and Kimura [16] hypothesized that sex differences in the object exchange task may be the result of women’s greater ability to process the identity of objects. The present study examines a corollary of this hypothesis: If females were indeed better at object exchanges due to a superior object identifications, then (relative to the male students) women ought to excel also in other task relying on object identity information such as the present scene task which manipulated object identity (but not object location).

## Method

### Ethics statement

This research has received ethics approval from the Flinders University Social and Behavioural Research Ethics Committee. Participants provided written informed consent. No minors or children were enrolled in the study. The Ethics Committee waived the parental consent on behalf of minors aged 17 years so that these participants were able to give written informed consent on their own.

### Participants

Sixty volunteers (30 males and 30 females) completed the study which was advertised on a computer noticeboard. The volunteers were university students and received course credit for their participation. The female students were aged between 17 and 48 years (mean: 23 years) and the male students were aged between 18 and 45 years (mean 21 years). Their first language was English.

### Materials

For the scene task, 40 computer-generated indoor and outdoor images of the interior and exterior of houses were presented for 20 seconds each on a computer screen. The images were constructed using the Architectural Design package, Home Design 4000 Version 1.0 by Punch Software. The images contained a minimum of 12 stimuli each. The Presentation software rescaled the images to be 785 x 500 (width x height) pixels, and the images were presented in landscape format in the centre of a monitor with a screen resolution of 1280 x 1024.

Participants were also given a subset of the Australian Council for Educational Research Co-operative Reading Comprehension Test [28].

### Procedure

Each participant was tested individually in the scene task and the vocabulary test. The scene task was presented first (see Hollingworth [29] for a similar procedure). It consisted of 4 practice trials (for students to become familiar with the task) which were followed by 40 experimental trials.

For each experimental trial the students studied an image for 20 seconds. At the conclusion of the 20 second study period a mask appeared on the screen for 1 second. The participant’s memory of the image was then tested in a two-alternative forced-choice memory test. In this memory test, two versions of the scene were shown consecutively (each for four seconds). Critically, only one of the versions corresponded to the original image. The alternative version was identical to the original scene with a single exception: one of the objects (the target object) was rotated or substituted with a similar object. In both versions, an orange arrow pointed out the target object. Presentation of the original and alternative scenes was separated by a 500 ms blank screen. Across the 40 experimental trials (20 rotations and 20 substitutions), the original scene was presented equally often as first or second choice.

After both scenes had been displayed for 4 seconds participants were prompted by a question mark in the middle of the screen to indicate which of the two versions corresponded to the image originally studied. This was done by pressing “1” or “2” on the keyboard. Note that participants were asked to base their choice on the target object indicated by the orange arrow (which constituted the only difference between the original and alternative versions). The next trial commenced after an interval of 1 second in which a cross hair was presented in the centre of the screen.

Half of the experimental trials were presented as described above. For the other half the memory test was altered such that no background information was available. In this background absent condition, only the target object was visible during the memory test (all other objects were removed).

Overall, the experimental trials consisted of 8 conditions which were the factorial combination of the following variables: background (present, absent), target object (rotated, substituted) and placement of original scene in the memory test (first, second). Each of the 8 conditions was presented by five different scenes, resulting in 40 experimental trials. Presentation of the scenes was randomised such that a given condition could not be presented more than twice consecutively.

At the conclusion of the scene task participants completed a 20 item subset of the ACER Reading Comprehension test. In the reading test, participants were shown 20 target words (presented in bold). For each word they had to select the word’s meaning from a choice of 4 words printed below the target word. The task measured their verbal ability. Females have been found to have an advantage for processing verbal stimuli [30] and it has been suggested that verbal ability has the potential to facilitate object location memory [31, 32].

Following the verbal test participants’ second and fourth digits were photocopied. Using a caliper, the photocopies were used to measure the lengths of the digits.

## Results

The scene task was analysed using a mixed analysis of variance (ANOVA) with sex as between-participants factor and background (present, absent), memory task (rotation, substitution) and placement (original scene: first, second) as within-participants factors. Response latencies on correct trials and error scores were analysed separately (see Table 1 for a summary of the main results). Preliminary analyses revealed that the RT and error data were not normally distributed. The data were transformed using a square root transformation (see Tabachnick & Fidell [33]) and re-analysed. The results of the transformed data mirrored that of the untransformed data. The untransformed data are presented here.

**Table 1:** Summary of mean scores for the scene task, vocabulary test and 2D:4D ratios. Standard errors are shown in parentheses.

	Scene Task			
	RTs (ms)	Errors	Vocabulary	2D:4D
Females	853 (60)	.93 (.08)	15.77 (.47)	.97 (.004)
Males	881 (60)	.99 (.08)	16.23 (.43)	.96 (.004)

The analysis of the latency scores revealed that females (mean: 853 ms, Std Error: 60 ms) and males (mean: 881 ms; Std Error: 60 ms) did not differ significantly in their response speed (*F*(1,58) = .10, *p* = .74). The two-way interactions between background and sex, *F* (1, 58) = 6.03, *p* = .01, *ηp* ^2^ = .09, and between background and memory task, *F* (1, 58) = 5.20, *p* = .02, *ηp* ^2^ = .08, were qualified by a significant three-way interaction between sex, background and memory task, *F* (1, 58) = 4.69, *p* = .03, *ηp* ^2^ = .08. This three-way interaction was due to males responding faster on the rotation than the substitution task when the background was present. In all other comparisons between the sexes that included background present and absent, substitutions were faster than rotations. The main effects of background (*F*(1,58) = .15, *p* = .69), memory task, *F* (1, 58) = 3.09, *p* = .08, and placement, *F* (1, 58) = 2.18, *p* = .14, did not reach statistical significance. None of the interactions involving placement was significant (*F*(1,58) < 1.50).

The analysis of error scores (number of errors out of 40 trials) also showed no significant differences (*F*(1,58) = .27, *p* = .60) between females (mean error score: .93, Std Error: .08) and males (mean error score: .99, Std Error: .08). Error scores were lower when the original scene was presented first (mean: .57, Std Error: .05) rather than second (mean: 1.35, Std Error: .10), *F* (1, 58) = 42.99, *p* < .001, *ηp* ^2^ = .42. Fewer errors were made for substitutions (mean: .86, Std Error: .07) than rotations (mean: 1.06, Std Error: .07), *F* (1, 58) = 6.75, *p* = .01, *ηp* ^2^ = .10. The main effect of background just failed to reach significance, *F* (1, 58) = 3.80, *p* = .056. The main effects of background and placement were qualified by a significant three-way interaction between sex, background and placement, *F* (1, 58) = 5.77, *p* = .01, *ηp* ^2^ = .09. Error scores were lower when the original scene was presented first rather than second for both females and males and irrespective of whether the background was present or absent. This difference was most pronounced for males in the background absent condition. The interaction between background, memory task and placement was found to be statistically reliable, *F* (1, 58) = 4.80, *p* = .03, *ηp* ^2^ = .07. While error scores were lower when the original scene was presented first rather than second, this effect was least pronounced for the substitution task in the background present condition. All other interactions were found not to be significant (*F*(1,58) < 2).

An independent t-test revealed that the female (mean: 15.77, Std Error: .47) and male (mean: 16.23, Std Error: .43) students did not differ on the vocabulary test, *t*(58) = −.72, *p* = .45. Likewise, females (mean: .97, Std Error: .004) and males (mean: .96, Std Error: .004) did not differ reliably in terms of their 2D/4D ratios, *t*(58) = .73, *p* = .50.

## Discussion

This study examined whether women excel in tasks relying on object identity information. In a computer-based task, participants were asked to identify which of two scenes they had studied previously. The identifications were based upon a single target object in a given scene, and the task thus required participants to recognize the identity of the target objects. The main finding was that the female and male university students tested did not differ in their performance in this task.

James and Kimura [16] investigated object location memory and observed sex differences favouring females for object exchanges, but no such effect for object shifts, a pattern of results which suggested that women were superior at identifying objects by comparison with remembering the location of objects. The present results are inconsistent with the assumption that females are better at remembering object identity information relative to males. In the scene task, the target objects were not moved, and in the alternative scene the object was either a substituted (similar) object or a rotated version of the original object. In other words, the forced-choice memory tested required object identity information only. Importantly, if females were better at object exchanges due to a superior ability to identify objects, then this superior ability should also have facilitated performance in the scene task examined here.

The statistical analyses included memory task (rotation and substitution) as a factor, allowing an examination of potential sex effects for the tasks. It is worthy of note that response errors were lower for substitutions than rotations (for both women and men). A similar effect was obtained for response latencies. This result is consistent with earlier findings that rotations are a cognitively effortful process [34, 35] which may have increased error rates for rotations relative to substitutions. The analyses also included placement (original scene: first, second) as variable. The results showed that error scores were lower when the original scene was presented first rather than second. It is possible that when presented with the original scene first participants were able to base their decision (which required identification of the original scene) on the first (original) scene alone. In contrast, presentation of the alternative scene first still required consideration of the second (original) scene, thereby increasing task difficulty and leading to higher error rates when the original scene was presented second.

It has been observed that verbal ability can facilitate object location memory [31]. Verbal ability is unlikely to have been a major factor affecting performance in the current experiment because the female and male student tested did not differ in the verbal ability task. Interestingly, the male and female students’ 2D:4D ratios were also found to be comparable (.97 for women and .96 for men). In other words, the women in our sample had unusually low 2D:4D scores such that the digit ratio of the female sample was similar to what is typically obtained for males [22]. It may therefore be argued that the female students had visuo-spatial abilities which were comparable to those of their male counterparts [23], possibly accounting for the lack of sex differences in the scene task.

James and Kimura [16] employed artificial stimulus arrays in which a multitude of objects were presented in a semantically unrelated manner. In contrast, this study employed more realistic computer-generated images of indoor and outdoor house scenes. The use of indoor and outdoor scenes allowed an examination of object identity effects in the context of stimuli which the participants were familiar with from their daily lives [21]. The absence of sex effects in object processing, which is not in agreement with James and Kimura, may indicate that the nature of the stimulus material can affect performance, an important issue for future work. Another possibility for the difference in results may be task demands. On the whole, in the current study both females and males made few errors, suggesting that the participants were able to perform the task without difficulty. It may be that James and Kimura’s task was more difficult, contributing to the difference in findings.

In conclusion, the present investigation examined a corollary of the hypothesis that sex differences in the object exchange task may be the result of women’s greater ability to process the identity of objects. Inconsistent with this hypothesis no evidence for a sex effect favouring females was found in the scene task, which relied entirely on object identity information. The nature of the stimuli presented (complex, realistic images of indoor and outdoor scenes), the difficulty of the task and the sample of female students tested (with average 2D:4D ratios of .97) are variables that may have affected performance and require examination in future studies.
